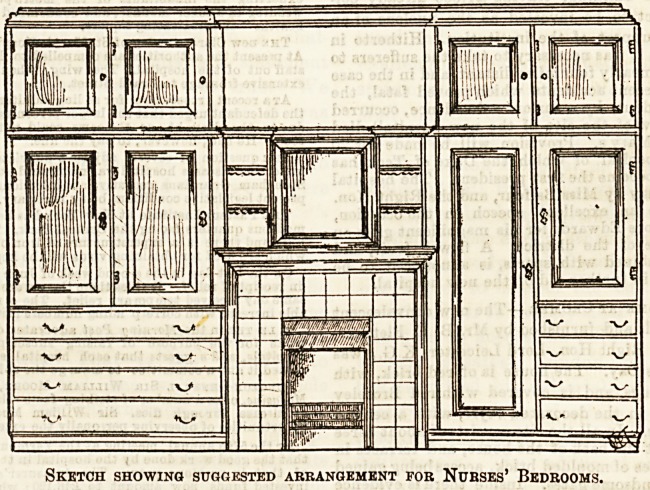# Furniture for Nurses' Rooms

**Published:** 1893-07-22

**Authors:** 


### PRACTICAL DEPARTMENTS.

### FURNITURE FOR NURSES' ROOMS.

A few weeks ago, in an article on this subject, we advocated "combination furniture" and fittings as being specially suitable for nurses' rooms. We are now able, by means of the accompanying little sketch, to give our readers an example of the arrangement suggested. As we mentioned before, the idea has been taken from a handbook on "Healthy Furniture and Decorations," published under the Council of the Health Exhibition of 1884, and written by Robert W. Edis, F.S.A. It seems to have many practical advantages, and we believe might well be given favourable consideration by hospital authorities. The illustration speaks for itself, and little comment is needed. The arrangement of cupboards, hanging cupboards and drawers, may of course be settled according to the shape of the room and position of bed, &c. The space over the fireplace can be fitted with glass, or the cupboard door panelled in the same way. In modern nursing homes, fireplaces are usually non-existent, all heating being done by means of hot water, and thus even more space would be available, and the position of the grate in our sketch might be used for table or wash-stand. A good idea would also be to provide a writing table, in the shape of a slab, to pull out for that purpose. This could easily be arranged for between the drawers and the cupboards on one side, and would be an immense convenience. Fittings of this kind, modified and adapted to suit the requirements of the rooms, would save much valuable space in the rooms opening from the wards, given to the sisters in some of our large hospitals, which have to do duty as bed and sitting-room combined, and where every available inch has to be made more use of than the uninitiated could imagine. In sitting-rooms the same plan can be carried out with book shelves and cupboards. In cases where it is impracticable for such fittings as these to be attempted, it is still possible so to arrange and design the moveable furniture as to ensure far greater convenience and comfort to the occupant of the room, and with no other expenditure than that of a little trouble and thought. In furnishing nurses' rooms it would surely be possible instead of crowding tiny rooms with three or four, more or less cumbersome articles, to combine as many uses in one as can be contrived. Thus the ordinary washstand to be found in such rooms occupies a certain amount of space, a good deal of which is simply wasted, as except for, generally speaking, a particularly narrow and inconvenient drawer, it can be used for nothing but the washing appliances. Now, the washstand should be provided with cupboards instead of, or in addition to the aforesaid drawer, which will be invaluable for the tidy storing away of boots and shoes, &c. The dressing table also should be made to combine its special purposes with those of a chest of drawers, and if instead of the ordinary wardrobe, a corner is utilized for that end, much room will be gained. Corner cupboards and washstands are an undoubted improvement, they prevent the harbouring of dust and of course save space. Messrs. Atkinson, Westminster Bridge Road, have much experience in institution furniture, and will carry out any designs that may be suggested in this way.

Sketch showing suggested arrangement for Nurses' Bedrooms.

## Figures and Tables

**Figure f1:**